# P-2327. West Nile Virus Disease in the Veterans Health Administration (VHA), 2010-2023

**DOI:** 10.1093/ofid/ofae631.2479

**Published:** 2025-01-29

**Authors:** Cynthia A Lucero-Obusan, Gina Oda, Joyce S Chung, Mark Holodniy

**Affiliations:** U.S. Department of Veteran Affairs, Public Health National Program Office, Palo Alto, California; Department of Veterans Affairs, Palo Alto, CA; VHA Public Health National Program Office, Palo Alto, California; Department of Veterans Affairs, Palo Alto, CA

## Abstract

**Background:**

Increasing evidence exists that West Nile Virus (WNV) transmission, distribution, and incidence is impacted by climate change. WNV is the most common mosquito-borne disease in the U.S., but there is little data in Veterans. Herein, we describe the epidemiology of WNV in VHA during 2010-2023.

**Figure:**
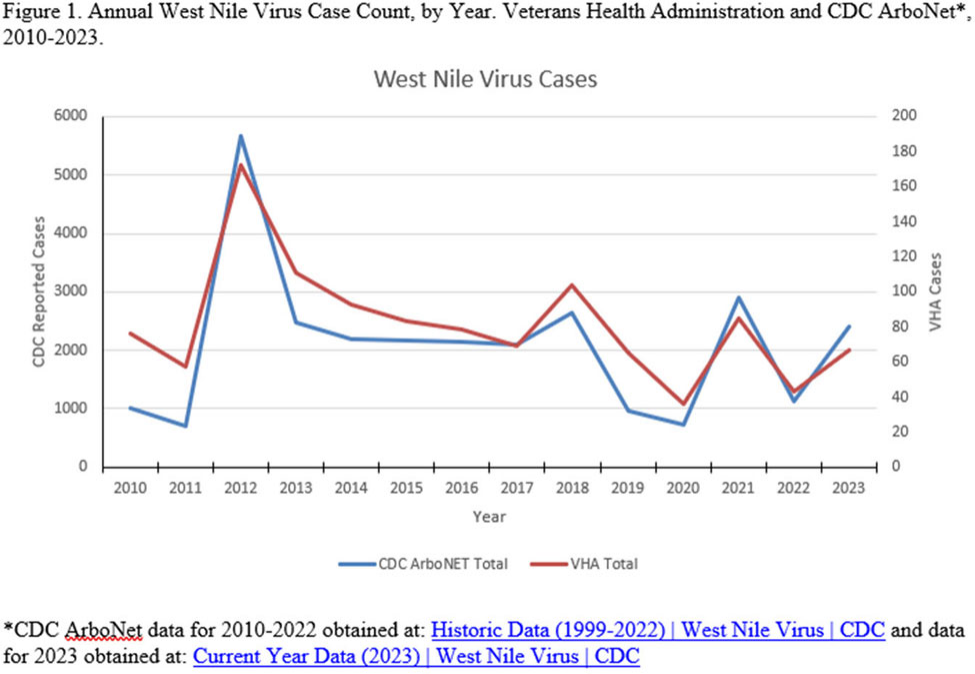

**Methods:**

WNV-coded hospitalizations, visits, and laboratory results were obtained from VHA data sources (1/1/2010-12/31/2023). We included individuals aged 18 years and above with ICD-9/10-CM WNV-coded encounters and/or a positive WNV IgM or PCR result. Data extracted included demographics and geography, diagnosis codes, test results, encounter details, and deaths during WNV-coded hospitalizations. Data were compared to CDC ArboNET.

**Figure:**
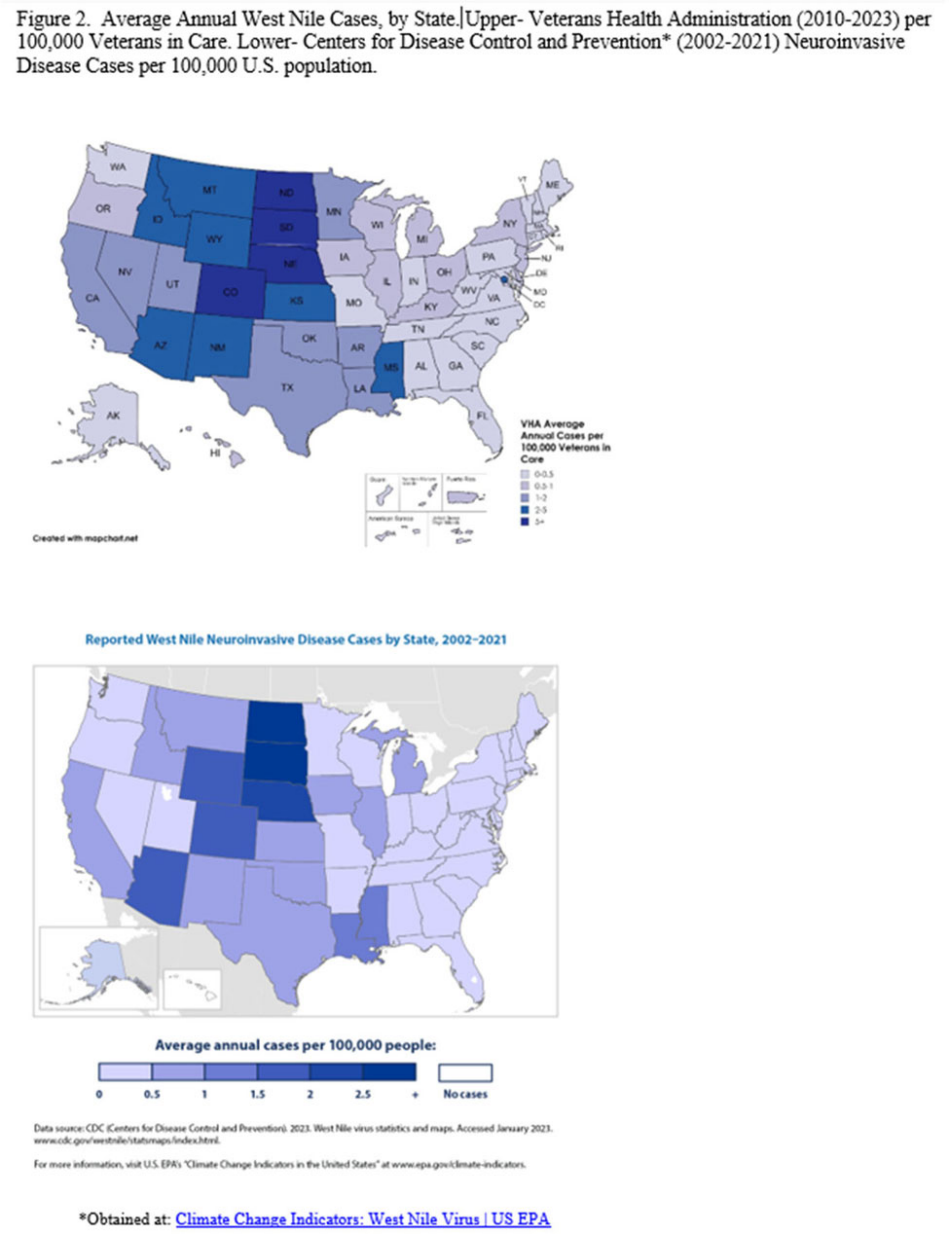

**Results:**

A total of 1,140 unique patients were identified. Of these, 762 (67%) were via ICD code only, 282 (25%) via ICD code & test result, and 96 (8%) via test result only. Annual case counts fluctuated but generally followed national trends, with highest activity observed in 2012, 2013 & 2018 (Fig. 1). Median age was 66 years and 92.4% were male. The highest proportion of cases were in males age 70+ (36%), which was also most common in ArboNET, although the percentage was lower (13%). 509 (44.6%) were coded as WNV encephalitis/neurologic manifestation and/or tested positive in the CSF, compared to ArboNET where 50.7% were classified neuroinvasive (1999-2022). Prevalence rates per 100,000 VHA population were highest for White (21.2) and American Indian/alaskan Native (AI/AN, 19.6) race and lowest in Asian (4.5). Average Annual case rates were highest in: South Dakota (16), Nebraska (11), North Dakota (6.6), and Colorado (5.1) (Fig. 2). There were 5,149 WNV outpatient visits (923 individuals). For 351 recorded hospitalizations (285 individuals), median stay was 11 days with 66 (18.8%) admitted to intensive care and 29 deaths during a WNV-coded hospitalization (8.3%).

**Conclusion:**

WNV causes substantial morbidity and the burden is likely underestimated as asymptomatic and mild cases may never seek care. VHA WNV activity tracks closely with national CDC ArboNET reporting. Additional targeted education may increase awareness and testing among at risk individuals, particularly in high incidence geographic areas.

**Disclosures:**

All Authors: No reported disclosures

